# Evaluation of Three Electronic Noses for Detecting Incipient Wood Decay

**DOI:** 10.3390/s100201062

**Published:** 2010-01-29

**Authors:** Manuela Baietto, Alphus D. Wilson, Daniele Bassi, Francesco Ferrini

**Affiliations:** 1 Dipartimento di Produzione Vegetale, Università degli Studi di Milano, Via Celoria, 2, 20133 Milan, Italy; E-Mail: daniele.bassi@unimi.it; 2 Southern Hardwoods Laboratory, Center for Bottomland Hardwoods Research, Southern Research Station, USDA Forest Service, P.O. Box 227, Stoneville, Mississippi, 38776, USA; E-Mail: dwilson02@fs.fed.us; 3 Dipartimento di Ortoflorofrutticoltura, Università di Firenze, Sesto Fiorentino (FI), Italy; E-Mail: francesco.ferrini@unifi.it

**Keywords:** tree hazard assessment, electronic aroma detection, wood-rotting fungi, urban landscape tree species

## Abstract

Tree assessment methodologies, currently used to evaluate the structural stability of individual urban trees, usually involve a visual analysis followed by measurements of the internal soundness of wood using various instruments that are often invasive, expensive, or inadequate for use within the urban environment. Moreover, most conventional instruments do not provide an adequate evaluation of decay that occurs in the root system. The intent of this research was to evaluate the possibility of integrating conventional tools, currently used for assessments of decay in urban trees, with the electronic nose–a new innovative tool used in diverse fields and industries for various applications such as quality control in manufacturing, environmental monitoring, medical diagnoses, and perfumery. Electronic-nose (e-nose) technologies were tested for the capability of detecting differences in volatile organic compounds (VOCs) released by wood decay fungi and wood from healthy and decayed trees. Three e-noses, based on different types of operational technologies and analytical methods, were evaluated independently (not directly compared) to determine the feasibility of detecting incipient decays in artificially-inoculated wood. All three e-nose devices were capable of discriminating between healthy and artificially-inoculated, decayed wood with high levels of precision and confidence. The LibraNose quartz microbalance (QMB) e-nose generally provided higher levels of discrimination of sample unknowns, but not necessarily more accurate or effective detection than the AromaScan A32S conducting polymer and PEN3 metal-oxide (MOS) gas sensor e-noses for identifying and distinguishing woody samples containing different agents of wood decay. However, the conducting polymer e-nose had the greater advantage for identifying unknowns from diverse woody sample types due to the associated software capability of utilizing prior-developed, application-specific reference libraries with aroma pattern-recognition and neural-net training algorithms.

## 1. Introduction

Tree instability in the urban environment is a major concern of public administrations whose principal aim is to assure that urban parks and gardens provide public settings that are beautiful, functional, clean, and above all, safe. Rots caused by wood decay fungi are the main causes of tree instability and associated hazards (*i.e.*, falling tree parts) because they commonly occur in many urban tree species and they weaken wood strength sufficiently to increase the chances of mechanical failure and catastrophic damage [1]. By destroying all components of wood cell walls (hemicelluloses, cellulose and lignin) via enzymatic degradation, wood-rotting fungi cause severe structural damage to wood within the trunk, main branches and root system of living trees [2]. As a consequence, trees often fail particularly during severe wind storms without showing any visual warning signs prior to failure.

Current tree-stability or hazard-assessment methods normally used in the urban environment involve a visual analysis of individual trees, referred to as Visual Tree Assessment (VTA) [3]. This analysis must be carried out by highly skilled and experienced personnel who must objectively evaluate all abnormal external characteristics of each tree that vary from typical healthy trees. An evaluation of the internal soundness of the tree, not discernable from visual assessments alone, is accomplished using various conventional specialized instruments. Many such instruments are available for internal assessments, although most are invasive, expensive, require skilled personnel, or are ineffective in the urban environment.

The detection and diagnosis of decays in trees using electronic-nose devices (e-noses) is based on the capability of detecting changes in the release of volatile organic compounds (VOCs) by either wood decay fungi or trees when wood decay is present. The composition of metabolites released by individual fungi is controlled largely by the types and combinations of metabolic pathways specific to microbial species which are regulated by genetic, substrate and environmental factors [4]. Korpi *et al.* [5] found microbes that released pinenes, acrolein, ketones and acetylenes that were irritants to mice. Other investigations have focused on the identification of VOCs released by food spoilage fungi [6,7]. The compound 1-octen-3-ol was detected in damp houses containing various mold fungi [8]. Numerous other chemical species have been reported as fungal metabolites, including complex acids, sesquiterpenes, methyl ketones and alcohols [9]. Relatively few recent studies have reported on the release of VOCs by healthy and decayed trees. An analysis of healthy *Populus* spp. and *Pinus* spp. indicated the presence of mainly monoterpenes, acetone and small amounts of isoprene [10]. Other studies have indicated increases in toluene and α-pinene emissions associated with *P. sylvestris* under pathogen attack [11], and a decrease in isoprene emissions from diseased *Quercus fusiformis* L. and *Q. virginiana* L. [12]. The bacteriostatic role of plant VOCs was studied by Gao *et al.* [13] who found emissions of terpenoids, alcohols, aldehydes, organic acids, and esters released by five healthy coniferous species in which α-pinene, β-pinene, 2,(10)-pinene, myrcene and d-limonene represented more than 95% of total VOC emissions. Increased levels of α-pinene, limonene, nonaldehyde and benzaldehyde also were found in artificially-inoculated wood shaves in the same study.

A living tree containing decayed wood releases a particular mixture of VOCs consisting of fungal metabolites, tree metabolites, and fungus-induced tree antimicrobial defense compounds (e.g., phenolic metabolites, terpenoids, isoprenoids, and phytoalexins). In order to quickly detect and discriminate changes in VOCs that are released by trees attacked by wood decay fungi, an instrument is needed that can electronically sense these changes in VOC emissions without having to identify the individual chemical species present in the volatile mixture. Such an instrument should be capable of detecting VOCs early in the decay process (at incipient stages), be non-invasive to the living tree, mobile, feasible for use in the urban setting and provide quick and effective determinations of internal decays caused by specific wood decay fungi. The objectives of this study were to (1) evaluate the capability of three different electronic-nose devices to discriminate between healthy and decayed wood and to (2) test the tentative feasibility of using these instruments for the early detection of incipient decays within living trees in the urban environment. The aim was not to directly compare the performance of the three e-noses, but to independently test the feasibility of each instrument because differences in detection mechanisms, data acquisition methods, run parameters, and data analysis methods (limited by the specific software designed and available to operate each instrument) precluded the use of the exact same analytical methods necessary for the acquisition of data directly-comparable between instruments. Some preliminary results from this study were reported previously [14,15].

## 2. Materials and Methods

This research was conducted over a three-year period (2005–2007) at the USDA Southern Research Station, Southern Hardwood Laboratory at Stoneville, Mississippi and at the Facoltà di Agraria of Milan laboratories using three different commercially-available electronic nose devices including: (1) the AromaScan A32S (Osmetech Inc., Wobum, MA, USA), an organic matrix-coated polymer-type 32-sensor array, (2) the “LibraNose 2.1” (Technobiochip, Pozzuoli, NA, Italy), a compact and semi-portable olfactory system with eight chemical quartz crystal microbalances sensors, and (3) the PEN3 (Airsense Analytics, Schwerin, Germany), a portable electronic nose provided with ten metal oxide semiconductor (MOS) sensors. These instruments operate based on entirely different operational mechanisms for VOC detection. Specific information concerning e-nose instrument design and detection principles is provided in the subheading descriptions for each individual instrument.

### 2.1. AromaScan A32S Electronic Nose

The AromaScan 32S is a conducting polymer electronic nose that contains an organic matrix-coated polymer-type 32-sensor array, designed for general use applications with 15 v across sensor paths. The sensor-array response to different VOCs was previously tested [4]. Sensors responses are measured as a percentage of electrical resistance changes to current flow in the sensors relative to baseline resistance (%ΔR/R_base_). The sorption of headspace volatiles, composed of specific VOC mixtures, to the conducting polymer sensor surfaces induce a change in the electrical resistance to current flow which is detected and measured to produce the sensor-array output. Sensor responses varied with the type of plastic polymer used in the sensor matrix coating, produced by electropolymerization of either polypyrrole, polyanaline or polythiophene derivatives, which have been modified with ring-substitutions of different functional groups and with the addition of different types of metal ions to the polymer matrix in order to improve and modulate sensor response. All measurements were statistically compared using normalized sensor outputs from the sensor array. The conducting polymer analysis (CPA) methods used with this instrument employ application-specific reference libraries for aroma pattern recognition and neural-net training algorithms.

#### 2.1.1. Samples Preparation

Headspace volatiles released from individual fungal cultures of twenty-four decay fungi, were analyzed individually (1–5 replications per sample type, based on availability) to test the capability of the AromaScan A32S to detect differences in fungal volatiles alone in pure culture. The wood decay fungi, including *Amylostereum areolatum* (Fr.) Boidin, *Armillaria gallica* Marxmüller and Romagnosi, *Armillaria mellea* (Vahl) P. Kumm, *Armillaria ostoyae* (Romagn.) Herink, *Armillaria tabescens* (Scop.) Emel, *Cerrena unicolor* (Bull.:Fr.) Murrill, *Daedalea quercina* (L.) Pers., *Daldinia concentric* (Bolton:Fr.) Ces. & De Not., *Fomitopsis officinalis* (Villars:Fr.) Bondartsev & Singer, *Ganoderma lucidum* (Curtis) P. Karst, *Gloeophyllum sepiarium* (Wulfen:Fr) P. Karst., *Hericium erinaceus* (Bull.:Fr.) Pers., *Heterobasidion annosum* (Fr.) Bref., *Inonotus dryadeus* (Pers. ex Fr.) Murr, *Laetiporus sulphureus* (Bull.) Murrill, *Oxyporus latemarginatus* (Durieu & Mont.) Donk, *Phanerochaete gigantea* (Fr.:Fr.) S.S. Rattan et al., *Phellinus pini* (Brot.) Bondartsev & Singer, *Pleurotus ostreatus* (Jacq.:Fr.) P. Kumm., *Postia caesia* (Shrad.:Fr.) P. Karst., *Spongipellis pachyodon* (Pers.) Kotlaba & Pouzar, *Stereum hirsutum*, (Willd.) Pers., *Wolfiporia cocos* (*F.A. Wolf) Ryvarden & R.L. Gilbertson*, and *Xylaria polymorpha (Pers.:Fr.) Grev.* were grown first on pure 4.5% Difco Malt agar (MA) culture medium 224200 (Becton Dickinson & Co., Sparks, MD, USA) in 100 × 15 mm-diameter plastic Petri dishes (Fisher Scientific, Pittsburgh, PA, USA) to make sure the cultures were pure without microbial contaminants. Pure axenic fungal culture samples for e-nose analysis were produced by transferring a single 5-mm plug of mycelium from a pure culture on Petri dishes into a 14.8 mL glass vials (21 × 70 mm) with screw-cap lids (Fisher Scientific), containing approximately 7 mL of the same MA medium, in order to allow the culture to grow and cover the 30°-slant agar surface over one to three weeks. The vials were capped, sealed and incubated at 25 °C ± 1 in the dark prior to analysis.

Wood samples from healthy trees were selected as controls for determinations of aroma signatures in the absence of decay. Tree bole sections 1–3 m long were collected from the trunks of nine tree species, including *Populus deltoides* Bartr. Ex Marshall, *Fraxinus pennsylvanica* Marsh., *Liquidambar styraciflua* L., *Quercus nuttallii* Palm., *Platanus occidentalis* L., *Quercus lyrata* Walt., *Thuja occidentalis* L., *Pinus taeda* L. and *Taxodium distichum* L., that were selected from among the hardwood and conifer species most common in the lower Mississippi Delta urban and forest environment. After one healthy tree of each species was cut down, the bole sections were placed in a desiccation room for about 24 hours at 60 C, peeled to remove the bark, and finally cut with a circular saw into 100 × 15 × 15 mm wood blocks (chip parallelepipeds). A minimum of 90 wood blocks from each tree was cut for a total of 810 wood block samples, and labeled using a felt-tipped marker. The samples were then sterilized in an autoclave at 121 °C for 15–20 minutes, dried in a 105 °C oven, then frozen at −20 °C in long-term storage until analyzed with each corresponding e-nose. These samples were considered “healthy” reference samples of which five replicate runs were analyzed for eight healthy wood types including: *Fraxinus pennsylvanica*, *Liquidambar styraciflua*, *Pinus taeda*, *Populus deltoides*, *Quercus lyrata*, *Quercus nuttallii*, *Taxodium distichum*, and *Thuja occidentalis*. These same control samples and decayed wood samples (fungus-host combinations) listed below were used in analyses for all three electronic noses, not just for the AromaScan A32S.

The “decayed samples” were produced by artificial inoculation of healthy wood blocks. A minimum of 3–6 wood blocks belonging to each of nine woody species (*Fraxinus pennsylvanica*; *Liquidambar styraciflua*; *Populus deltoide*s; *Plantanus occidentalis*; *Pinus taeda*; *Quercus nuttallii*; *Quercus lyrata*; *Taxodium distichum*; and *Thuja occidentalis)* was inoculated separately with 11 different wood decay fungi, including: *Armillaria gallica*, *Armillaria mellea*, *Armillaria ostoyae*, *Armillaria tabescens*, *Daedalea quercina*, *Ganoderma lucidum*, *Heterobasidion annosum*, *Inonotus dryadeus*, *Laetiporus sulphureus*, *Phellinus pini* (Thore:Fr.) A. Ames, and *Stereum hirsutum*. Wood blocks of different host woods (*Fraxinus pennsylvanica*; *Liquidambar styraciflua*; *Populus deltoide*s; *Plantanus occidentalis*; *Pinus taeda*; *Quercus nuttallii*; *Quercus lyrata*; *Taxodium distichum*; and *Thuja occidentalis*) were artificially inoculated by dipping them into inoculum of each fungus using the following procedures. All of the isolates were previously preserved in sterile H_2_O as mycelial plugs, in the USDA Pathology Lab of the Southern Research Station of Stoneville, within 1.8 mL cryotubes (Nunc A/S, Roskilde, Denmark) stored in a 5 °C refrigerator [16]. Two plugs of mycelium were transferred from the cryotubes to sterile Petri plates containing 4.5% Malt agar M9802 (Sigma-Aldrich, St. Louis, MO, USA). The substrate was previously sterilized for 40 min in an autoclave at 121 °C and 15 psi, then poured into 10 cm Petri plates and cooled down to room temperature on a sterile surface within a laminar flow hood (Nuare Laminar Flow Products, Plymouth, MN, USA). Each of the two mycelium plugs was spatially placed far apart on the same Petri dish in order to obtain the widest culture margin possible between the fungal colonies. Every Petri dish was firmly sealed with Parafilm “M” (Pechiney Plastic Packaging, Chicago, IL, USA). In order to obtain pure cultures and to prevent further bacterial proliferation, all the isolates were transferred a second time to new sterile dishes containing 4.5% Malt agar medium. Eight mycelial plugs from each Petri dish culture were transferred into a 250 mL Pyrex^®^ sterile glass flask with 150 mL of 3% Malt Extract (Merck KGaA, Darmstadt, Germany) sterile broth. The flasks were then plugged with sterile cotton and gently shaken in order to distribute the plugs in the flasks. After one to three weeks, a large mass of mycelium was formed into the flasks. A hand-held stainless steel mixer (T10 basic Ultra-Turrax^®^, IKA^®^ Werke GmbH & Co. KG, Staufen, Germany) with previously autoclaved blades was used to macerate the mycelial mass within the liquid culture. Inoculated wood blocks were incubated at 25 °C ± 1 in the dark for 12 months resting on 4.5% Malt agar substrate. After incubation, they were removed from the tubes, rinsed with tap water in order to remove every visible trace of fungal mycelium, blotted on tissue paper, wrapped in aluminum sheets, and stored at 5 °C.

#### 2.1.2. Pre-Run Procedures and Data Collection

Healthy samples that became desiccated during storage were rehydrated by soaking in sterile distilled water for 15 minutes, followed by blotting on tissue paper to remove excess free moisture immediately prior to analysis. Healthy, decayed and pure culture samples were placed into a 500-ml Pyrex glass Laboratory storage sampling-bottle 1395 (Corning Inc., Corning, NY, USA), fitted with reference air, sampling, and exhaust ports on a polypropylene bottle cap, and firmly sealed to let the volatiles build headspace and equilibrate for 30 minutes prior to each run. The sampling bottle was held in the sampling chamber within the instrument at a constant air temperature of 25 °C ± 0.5 for the entire run period.

All pre-run procedures, instrument configurations, run parameters, data-acquisition settings, and run schedules followed the method specifications used by Wilson *et al.* [4]. Pre-run tests were performed to determine sample air relative humidity compared with that of reference air. Reference air was set at 4% relative humidity and adjusted to within 2% below sample air at 25 °C. The temperature of the sensor array was maintained at a constant 30 °C. Reference air was preconditioned by passing room air sequentially through a carbon filter with activated charcoal 05-685B (Fisher Scientific), silica gel beads (EMD Chemicals Inc., Gibbstown, NJ, USA), inline pump-protection filter 0500-0006 (Osmetech Inc., Wobum, MA, USA) and glass microfiber Hepa-Cap™ 36 filter (Whatman Inc., Clifton, NJ, USA) to remove organic compounds, moisture, particulates, and microbes, respectively. The flow rate (vacuum) of sample air at the sampling port was maintained at −700 ml/min using a calibrated ADM 3000 flow meter (Agilent Technologies, Wilmington, DE, USA). Sensors were purged between runs using a noncommercial 2% v/v isopropanol wash solution. The instrument was interfaced with a personal computer via a RS232 cable and controlled with AromaScan 3.51 software (Osmetech Inc.).

A uniform run schedule was used as following: reference air, 20 s; sampling time, 90 s; wash, 20 s; reference air, 90 s; a 2 min reference air purge followed by a 30 min equilibration period between the runs. Data from the sensor array were collected at 1 s intervals, and a conventional 20-s sampling interval (between 85 and 105 sec into the run) near the end of the sampling segment was used [4].

#### 2.1.3. Construction of Reference Libraries, Validation and Statistical Analyses

For each specific category of samples (healthy, partially-decayed wood blocks and decay fungi in pure cultures), separate reference libraries were constructed from known samples of each sample category (aroma class) included in each reference library [4]. All database files of reference types were linked to designated aroma classes for each sample type. A separate neural-net training session was conducted to create a unique reference library for each sample type. A minimum of eight replicate runs per sample category were used to build each application-specific reference library database. Neural net trainings were validated by examining training results that compared individual database files for compatibility with each defined aroma class, and showed similarity matches using aroma class distributions among all aroma classes included in the recognition files created by the training.

Unknown samples were identified by comparison of the response pattern with application-specific reference libraries for each sample type (fungi in culture, healthy wood, and decayed wood). The pattern-recognition algorithms determined the aroma profile that most closely fit the aroma elements found in the unknown sample, recorded as a percentage value (mean % aroma class match) allocated to different global aroma classes represented in the sample. A value greater that 90% was considered sufficient to judge an unknown sample as adequately recognized. A significance level of α = 0.05 was used to train the neural net. Detailed comparisons of relatedness of odor classes were determined using principal component analysis (PCA) algorithms provided by the AromaScan Version 3.51 software.

### 2.2. Libranose 2.1 Electronic Nose

This electronic nose is based on the quartz crystal microbalance technology (QCM) which can be described as an ultrasensitive sensor capable of measuring small changes in mass on a quartz crystal recorded in real-time. The heart of the quartz crystal microbalance is the piezoelectric AT-cut quartz crystal sandwiched between a pair of electrodes. The electrodes are attached to an oscillator. When an AC voltage is applied over the electrodes, the quartz crystal starts to oscillate at its resonance frequency due to the piezoelectric effect. If sample volatiles are evenly deposited onto one or both of the electrodes, the resonant frequency will decrease proportionally to the mass of the adsorbed layer according to the Sauerbrey equation [17]. LibraNose 2.1 sensor array consists of eight 20 MHz AT-cut quartz crystal microbalance sensors with a gold surface (Gambetti Kenologia, Binasco, PV, Italy) coated with either metalloporphyrines (Table 1), deposited by solvent casting, or by polypyrrole polymer (Table 2) films (Technobiochip patent. no. 04425560.2-2102) deposited by means of Langmuir-Blodgett technology using a KSV 5000 film-deposition device (KSV Instruments, Helsinki, Finland). This process utilizes 0.3 mg/mL polymers dissolved in chloroform and ultrapure, distilled water as a subphase.

#### 2.2.1. Samples Preparation

A single shade tree of the following ten species (including *Acer negundo* L., *Acer saccharinum* L., *Aesculus hippocastanum* L., *Castanea sativa* Mill., *Cedrus deodara* (D. Don) G. Don fil., *Celtis australis* L., *Platanus* x *acerifolia* Brot., *Quercus rubra* L., *Robinia pseudoacaci*a L., and *Tili*a spp.) was cut down in the urban or peri-urban northern Italian environment, peeled to remove the bark, and cut into wood block parallelepipeds. A minimum of 96 wood blocks were cut from each tree species, sterilized in an autoclave (into a 50 ml capped self-standing polypropylene test tube (M. Medical, Milan, Italy) at 121 °C for 20 minutes and dipped into a previously prepared liquid Difco potato-dextrose (PD) broth culture medium 254920 (Becton Dickinson & Co.) containing inoculum of one of ten wood decay fungi chosen for the study (*Armillaria gallica*, *Armillaria mellea*, *Armillaria ostoyae*, *Daedalea quercin*a, *Ganoderma lucidum*, *Heterobasidion annosum*, *Inonotus dryadeus*, *Laetiporus sulphureus*, *Phellinus pini*, and *Stereum hirsutum*). The wood decay fungi were isolated from tissues of freshly collected basidiocarps. These were collected near or at the base of the stems from living trees with much decay, so as to be assured of good decay potential of the fungi collected. Pure cultures were obtained by placing a 2-mm piece of contextual mycelium from each fresh fruiting body onto 4.5% Malt agar medium containing 0.1% (w/v) streptomycin sulfate S6501 (Sigma-Aldrich). Mycelial plugs of each pure-culture isolate placed in long-term storage were later transferred at least two times on the same substrate in sterile Petri dishes of 4.5% Malt agar before the liquid broth cultures were finally prepared.

The inoculated wood blocks were incubated in 25 °C ± 1 in the dark for 12 months (decayed samples). Sterilized wood blocks dipped into sterile liquid PD broth were used as controls (healthy samples). After the incubation period, the wood blocks were rinsed with tap water to remove visible traces of fungal mycelium and blotted on tissue paper. A minimum of eight replicate samples per analyte species (wood-fungus combinations plus controls) were prepared for this study. A total of 60 different analyte species and 480 total samples were analyzed by the LibraNose electronic nose.

#### 2.2.2. Prerun Procedures, Data Collection and Statistical Analyses

Immediately prior to sample analyses by the electronic-nose device, the samples were blotted by means of blotting tissue to remove excess moisture and put into an uncapped 500 mL Pyrex glass laboratory sampling bottle 1395 (Corning Inc.) firmly wrapped with Parafilm to allow volatiles from the samples to build headspace and equilibrate for 60 minutes prior to each run. All measurements were performed at 30 °C (sensor array chamber temperature) and atmospheric air was used as the carrier gas. Reference air was preconditioned by passing room air sequentially through a series of filters to remove organic compounds, moisture, particulates and microbes. The flow rate (suction) of sample air at the sampling port was maintained at 600 mL/min by an automatic pump to aspirate the headspace and pass it over the sensors. Sensors were purged between runs using filtered and conditioned room air. The LibraNose 2.1 instrument was controlled by E-nose software (Sigeda, Milan, Italy). The total measuring cycle for each sample was 30 min. A uniform run schedule was used. Data from the sensor array were collected at 1 s intervals and the averaged data of three repetitions per sample were taken per run during data acquisition. Principal component analysis (PCA) was performed to reduce the responses of the eight sensors to two principal components. This tool did not provide any sample recognition, but only sample discrimination by principal components. Reference libraries were not used for sample recognition and data analyses with the Libranose e-nose.

### 2.3. PEN3 Electronic Nose

The PEN3 electronic nose is a very compact instrument (255 × 190 × 92 mm), light-weight (2.1 kg) and portable olfactory system. It consists of an array of 10 different doped semi-conductive metal-oxide gas sensors (MOS) positioned into a very small chamber with a volume of only 1.8 mL. The instrument operates with filtered, ambient air as a carrier-gas at a flow rate of 10–400 mL min^−1^, sample-chamber temperature of 0–45 °C, and sensor-array operating temperature of 200–500 °C. The sensing reaction is based on an oxygen exchange between the volatile gas molecules and the metal coating material. Electrons are attracted to the loaded oxygen and result in decreases in sensor conductivity. Instrument sensitivity to various gas analytes ranges from 0.1–5.0 ppm. Table 3 lists all the PEN3-sensors used in the experiment and their sensitivity to specific analytes.

#### 2.3.1. Samples Preparation

Wood samples were prepared for aroma plot analysis using two principal components based on linear discriminant analysis (LDA) of volatiles from healthy and decayed wood blocks of eight host woods (*Acer negundo*, *Acer saccharinum*, *Castanea sativa*, *Cedrus deodara*, *Celtis australis*, *Platanus x acerifolia*, *Quercus rubra* and *Robinia pseudoacacia*) after 12 months of decay by five different wood decay fungi: *Armillaria mellea*, *Armillaria ostoyae*, *Ganoderma lucidum*, *Heterobasidion annosum* and *Inonotus dryadeus*. A minimum of 3 replicate samples of each fungus-host wood combination and healthy wood (controls) were prepared and analyzed by the PEN3 e-nose in this study. All possible combinations of host woods and wood decay fungi were tested. Wood block test samples were prepared using the same wood collection, inoculation, incubation and post-decay procedures applied for the AromaScan A32S and LibraNose 2.1, except that the decay fungus-host wood combinations and total number of samples prepared for analysis were different. The headspace volatiles of *Aesculus hippocastanum* wood decayed by four different wood decay fungi, including *Armillaria mellea*, *Inonotus dryadeus*, *Ganoderma lucidum*, and *Heterobasidion annosum*, also were prepared to distinguish between healthy and decayed wood using principal component analysis (PCA).

#### 2.3.2. Pre-Run Procedures, Data Collection and Statistical Analyses

The instrument was pre-warmed for 10 minutes at the beginning of each run session. A uniform run schedule was used for all samples based on the following run cycle: sensors cleaning, 300 s; sampling run time, 80 s. Data from the sensor array were collected at 1 s intervals and all data were averaged from three replications per sample. A conventional 10-s sampling interval between 38 and 48 sec into the run was utilized. A luer-lock needle (Terumo Italia srl, Rome, Italy) connected to 3 mm Teflon-tubing (Fisher Scientific) was used to perforate the wrap of each bottle and to aspirate the sample air with accumulated headspace volatiles. The headspace gas was pumped over the sensors at a carrier gas flow rate of 400 mL min^−1^ and the run cycle was controlled by Winmuster 1.6.2.5 software (WMA Airsense Analytics GmbH).

The sampling chamber temperature was set at 30 °C, controlled by thermostatic bath, and atmospheric air was used as the carrier gas. Reference air was preconditioned by passing room air sequentially through an active-carbon filter (Whatman plc, Maidstone, UK) to remove organic compounds, moisture, particulates and microbes. Because sensor conductivity drifted as the sample gas passed over the array, the data were adjusted based on the changing ratio of conductivity between G and G_0_ (*i.e*., the electrical conductivity response of the sensors to the sample gas relative to the carrier gas or baseline signal over time).

All samples were analyzed at least two times and the mean values between averaged sensor values were used for statistical analysis, as well as the peak value of all sensors. Principal component analysis (PCA) and linear discriminant analysis (LDA) were performed by Winmuster software to discriminate between the different classes of samples. PCA allowed the extraction of useful information from the data and to explore their structure (including correlation between variables and the relationship between the subjects), whereas LDA maximized the variance between the sample categories (aroma classes) and minimized the variance within the same category or class [19]. Reference libraries were not used for sample recognition and data analyses with the PEN3 e-nose analyses.

## 3. Results

### 3.1. AromaScan A32S Electronic Nose

**Figure 1.**
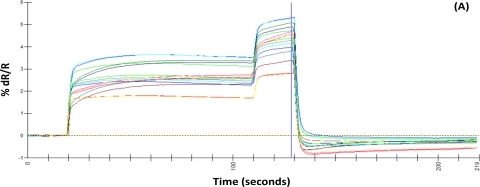

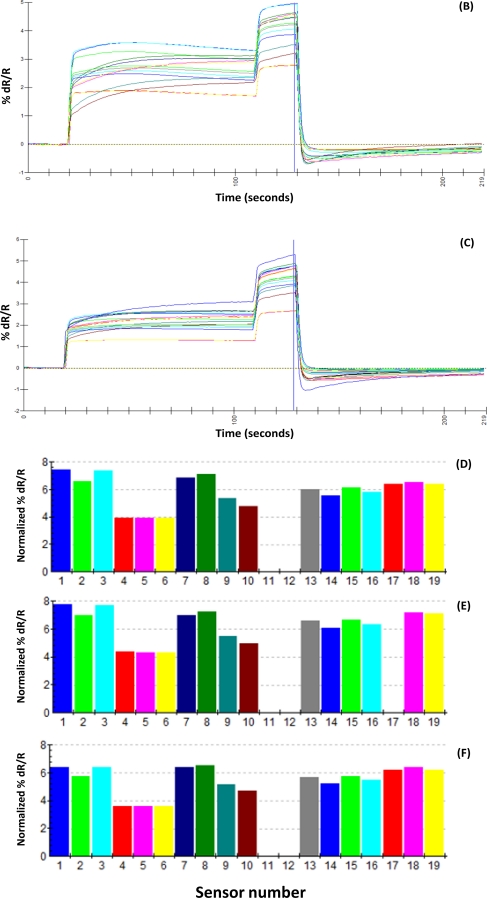
Typical sensor-response outputs from representative samples of healthy wood blocks (controls), inoculated wood blocks (decayed) and wood decay fungi pure cultures. Sensory array output are from: (A) a non-inoculated sample of *Quercus lyrata*, (B) a partially decayed wood sample of *Q. lyrata* after inoculation with *Ganoderma lucidum*, and (C) a sample of *Ganoderma lucidum* in pure culture. Histograms of normalized intensity responses of individual sensors to headspace volatiles produced by the same *Q. lyrata*-samples for non-inoculated (D), partially decayed (E), and pure culture (F), respectively.

Typical sensor output graphs for representative sample types using the AromaScan A32S are presented in Figure 1, A–C. Headspace volatiles from healthy (non-inoculated), decayed (inoculated) wood blocks and wood decay fungi in pure culture produced relatively similar response curves with 5–6% change in electrical resistance for individual sensors.

Comparisons of normalized sensor outputs expressed as histograms (Figure 1, D–F) indicate high variability between sensors responses to volatiles and relatively lower sensor responses to volatiles from wood decay fungi cultures compared with healthy and decayed wood samples. Sensors 11, 12 and 20–32 did not add significantly to the discrimination of aroma classes and were excluded from the analysis.

Three aroma classes, utilized for recognition of unknown samples, included pure cultures, healthy, and decayed wood samples, respectively. Most unknown samples (93.2%) of the 24 wood decay fungi analyzed on pure MA culture medium were identified correctly to the right aroma class (Table 4). Only 6.8% of sample unknowns, including only cultures of *Inonotus dryadeus* and *Phellinus pini*, were indeterminate or unidentified. The mean % aroma class match ranged from 63 to 100%. Matches greater than 80% generally are considered sufficient for reliable identifications when combined with PCA data. Almost one-third of unknowns correctly identified had a mean % aroma class match above 80%. Matches less that 80% usually were due to insufficient replications per sample. The decay fungi with the lowest level of mean % aroma class match were *Stereum hirsutum*, *Pleurotus ostreatus*, *Wolfiporia cocos* and *Armillaria ostoyae.* Three fungi (*P. gigantea*, *A. mellea*, *and G. lucidum*) had the highest % aroma class match. None of the decay fungi sample unknowns were incorrectly identified.

A large proportion (80%) of uninoculated wood type unknowns was correctly identified as “healthy”, 10.0% incorrectly identified (as “decayed”) and 10.0% were unidentified or indeterminate (Table 5). The lowest level (60%) of correct identifications occurred with wood samples of *Liquid ambar styraciflua* and *Taxodium distichum.* The mean % aroma class match for healthy wood types ranged from 75.3% to 95.2%. The unknown wood types with the lowest level of mean % aroma class match were *Pinus taeda* and *Liquidambar styraciflua*. All other wood types had good levels of discrimination with mean % aroma class matches greater than 86%. These levels of discriminations were achieved with a sampling rate of only five replications per unknown wood type.

The majority (95%) of all decay type unknowns, based on decay by nine wood-rotting fungi, from among eleven wood types analyzed were correctly identified (Table 6). Of 378 artificially-inoculated wood blocks (decayed), only 3.7% were unidentified and only 1.3% were incorrectly identified. Wood types decayed by *Laetiporus sulphureus* were the least identifiable as decayed (11.1%) among all wood decay fungi tested. All wood species decayed by *Armillaria gallica* were correctly identified as “decayed”, except for one sample of *Taxodium distichum*, which was misidentified as “healthy”. Among *Armillaria melle*a decayed samples, only one sample each of *Populus deltoides* and *Pinus taeda* was not identified. *Armillaria ostoyae* and *A. tabescens* decayed wood samples also were identified at high levels with only one sample in each case not identified. Identifications of wood decayed by *Daedalea quercina* were similar with only one sample of *Quercus nuttallii* not identified. The decay type of *Heterobasidion annosum* and *Stereum hirsutum* were very effectively identified with 100% of host wood samples analyzed correctly identified as “decayed” by these respective decay types. The mean % aroma class match for decay type unknowns ranged from 85.0 to 95.6%.

*Tilia* sp. wood samples displayed the greatest differences between healthy wood and five decay types as indicated by discrimination between sample analytes at very high levels of significance using pair-wise PCA (Table 7). These high levels of discrimination between healthy and decayed woods of *Tilia* sp. by five different wood decay fungi were determined with exceptionally high Quality factor (QF) values indicating statistically-significant differences at P < 0.001 or lower. The highest levels of discrimination were found between healthy *Tilia* sp. wood and wood decayed by *Ganoderma lucidum* and *Heterobasidion annusum*.

### 3.2. LibraNose 2.1 Electronic Nose

**Figure 2.**
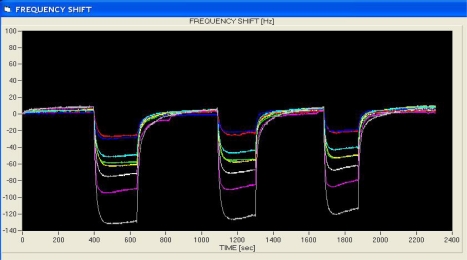
LibraNose 2.1 e-nose sensor-array output derived from *Ganoderma lucidum-*decayed *Celtis australis* wood block one year after artificial inoculation. Three replication analyses were performed per sample during a 2,400 s run cycle. Notice that there is a complete reversal of sorption of volatiles to the sensors as indicated by frequency shifts at three separate instances during the run.

A typical sensors output signal from the quartz microbalance transducer of the LibraNose 2.1 e-nose is presented in Figure 2. The output sensor signals are for volatiles from a *Ganoderma lucidum-*decayed wood sample of *Celtis australis* one year after artificial inoculation. Three replication analyses were done on the same sample during the run. It is very clear how the sensors-odor vapor bonds are completely reversible as indicated by the base frequency oscillation when it returns to zero. When the headspace volatiles adsorbed to the polypyrrole polymers or metalloporphyrines in the sensor array, the quartz system detected that the crystals were heavier and thus oscillated more slowly. Changes in the frequency of quartz crystal oscillation (Δf) provide the means for detecting differences in component headspace volatile mixtures present in the analyte sample. The oscillation frequency of the quartz crystals change (undergo a frequency shift) when VOC compounds adsorb to the sensor surface resulting in a change in weight on the crystals which modulates vibration frequency that is detected by the transducer and recorded on the sensor-array output.

**Figure 3.**
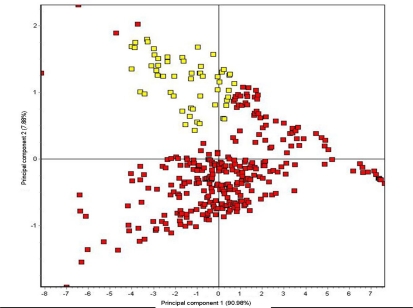
Aroma map plot showing discrimination of volatiles from healthy control wood blocks (yellow labels) and volatiles from artificially-inoculated decayed wood block (red labels) using principal component analysis (PCA) of mean changes (shifts) in quartz crystal oscillation frequency (Δf) in response to differences in VOC mixture composition in headspace volatiles from different sample types.

The capability of the LibraNose 2.1 electronic nose to discriminate between uninoculated healthy control and decayed samples was tested first with ten healthy wood types compared with these same woods decayed by nine different wood decay fungi replicated at least two times. Figure 3 demonstrates how volatiles from the healthy control wood samples (yellow labels) were clearly separated from volatiles of artificially-inoculated decayed wood blocks (red labels). Data analyses using PCA indicated that principal component 1 and principal component 2 explained 91.0% and 7.9% of the sample variance, respectively. The LibraNose 2.1 clearly distinguished decayed samples from healthy or control samples with good precision and high levels of discrimination. Four outlying data points for decayed wood samples occurred outside of the majority clustering of data points for most of the decayed wood types, but not overlapping with the data point cluster for healthy woods. This outlying data represent headspace volatiles specifically produced by *Armillaria ostoyae* decay of *Acer saccharinum* wood and account for only 1.9% of the data points represented by the decayed wood types (n = 215), and only 1.5% of all samples analyzed (n = 270). The volatiles associated with decay in this incidence are unique and most likely explained by a different complement of secondary metabolites released by *A. ostoyae* on this particular woody substrate. Substrate-induced variations in microbial metabolic pathways are not uncommon, particularly with wood decay fungi.

The possibility of discriminating between different etiologic agents (decay fungi) of wood decay was investigated in a separate analysis. Principal component statistical analysis of changes in mean crystal-oscillation frequency for healthy and decayed wood of *Celtis australis*, *Tilia* spp., and *Acer negundo* wood types were tested, respectively.

**Figure 4.**
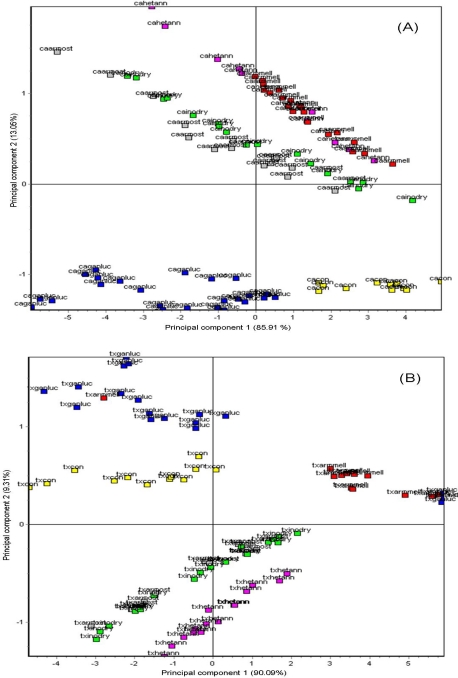

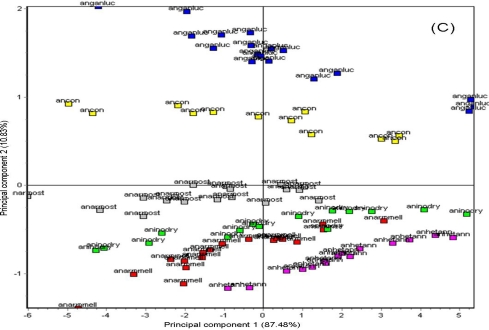
Aroma map plot showing discrimination of volatiles from decayed wood samples of (A) *Celtis australis*, (B) *Tilia* spp. and (C) *Acer negundo* by principal component analysis (PCA) of mean changes in crystal oscillation-frequency (Δf) data. Different color labels^1^ indicate different wood decay fungi responsible for decay and corresponding headspace volatiles produced. The first two letters of each label indicate specific tree species according to the following abbreviations: ca = *Celtis australis*; tx = *Tilia* sp.; an = *Acer negundo*. The subsequent letters following in the label indicate the wood decay fungus responsible for decaying the wood blocks for 12 months following artificial inoculation: ganluc = *Ganoderma lucidum*: hetann = *Heterobasidion annosum*; armmell = *Armillaria mellea*; armost = *A. ostoyae*; inodry = *Inonotus dryadeus*. Con = undecayed or healthy (control) wood blocks.

Results of comparisons between artificially-inoculated wood decayed by five wood decay fungi and uninoculated healthy controls are presented in Figure 4 A–C. Healthy undecayed woods (yellow labels) were clearly separated from decayed woods for all three wood types. Moreover, differences in PCA clustering of wood decayed by different wood decay fungi generally indicated clear separations of PCA groupings of each fungus-wood type combination, demonstrating effective discriminations between different types of decay caused by specific decay fungi in different host woods and between decayed woods from healthy woods of these three hosts.

PCA analysis of decay types in *Celtis australis* wood showed that *Ganoderma lucidum*-decayed wood samples were well separated from decay types caused by the other wood-rotting fungi (Figure 4A). However, *Heterobasidion annosum*–decayed wood samples were not clearly separated from *Armillaria mellea*-decayed wood, and *Inonotus dryadeus*-decayed wood was in the proximity and not well separated from *Armillaria ostoyae*-decayed wood of *C. australis*.

PCA indicated that principal component 1 and principal component 2 explained 85.9% and 13.1% of the sample variance, respectively for the *Celtis australis* sample plot. The results of PCA tests in the *Tilia* spp. wood type were quite different (Figure 4B). *Ganoderma lucidum*-decayed wood samples were more widely scattered and had some minor integration with *A. mellea*-decayed wood although the bulk of the data clusters were well-separated for these two sample types. *Inonotus dryadeus*-decayed wood was closely associated with and had significant integrated clustering with *A. ostoyae*-decayed wood. However, *H. annosum*-decayed wood and healthy control samples had well-separated data-clustering, indicating effective discrimination from the other sample types. PCA indicated that principal component 1 and principal component 2 explained 90.1% and 9.3% of the sample variance, respectively for the *Tilia* spp. sample plot.

Data-clustering patterns for decay types, based on PCA tests of the *Acer negundo* wood type, were much more clearly separated (Figure 4C). *Ganoderma lucidum*-decayed wood was the most widely separated from the other four wood decay types and healthy undecayed controls. *Inonotus dryadeus*-decayed wood was in close proximity to *A. mellea*-decayed wood with some minor integration, but still fairly-well discriminated for most of the data clusters. *Armillaria ostoyae*-decayed wood samples were clustered in a location well separated from the other decay types in this host wood. PCA indicated that principal component 1 and principal component 2 explained 87.5% and 10.8% of the sample variance, respectively for the *Acer negundo* sample plot.

### 3.3. PEN3 Electronic Nose

The PEN3 instrument is a MOS-type electronic nose with a sensor array composed of ten sensors that are coated with semi-conducting metal oxide. Metal oxide semiconductor sensors consist of three layers: a silicon semiconductor, a silicon oxide insulator and a catalytic metal through which the applied voltage creates an electric field. The sorption of gas molecules provoke changes in conductivity brought about by combustion reactions with oxygen species on the surface of the metal-oxide sensors. When polar compounds interact with the metal, the electric field is modulated and recorded by the transistor.

**Figure 5.**
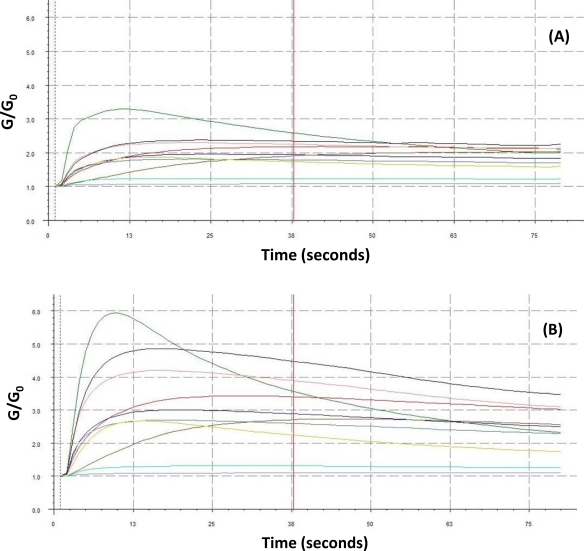

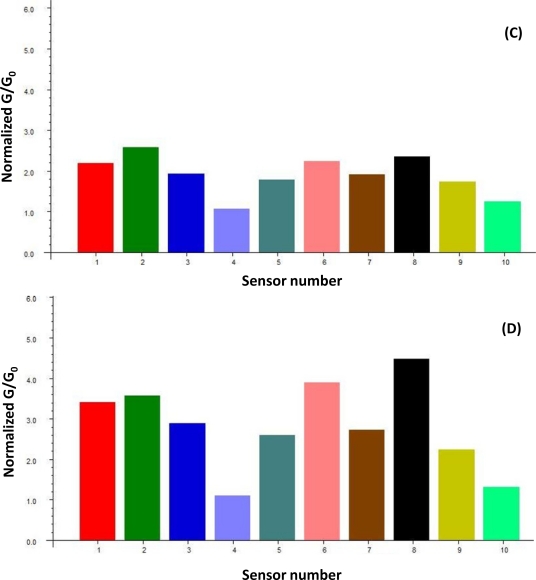
Typical sensor-response outputs from representative samples of healthy wood blocks (controls), and inoculated wood blocks (decayed). Sensory array output from: (A) a non-inoculated sample of *Acer saccharinum*, (B) and a decayed wood sample of *A. saccharinum* after inoculation with *Armillaria mellea*. Histograms of normalized intensity responses of individual sensors to headspace volatiles produced by the same *Q. lyrata*-samples for non-inoculated (C), and decayed wood (D), respectively. G/G_0_ is the ratio of the conductivity response of the sensors to the sample gas (G) relative to the carrier gas (G_0_) over time. The following data were adjusted relative to the carrier gas baseline.

Typical PEN3 sensor output graphs for representative healthy and decayed wood sample types are presented in Figure 5 A–B. The graphs show comparisons between the sensors signals of one healthy (non-inoculated) and one decayed (inoculated) wood block after one year of decay for a sample of *Acer saccharinum* and *Acer saccharinum*, respectively, previously inoculated with the wood decay fungus *Armillaria mellea*. Comparisons of normalized sensor outputs expressed as histograms are presented in Figure 5, C–D. The highest variability between sensors responses to volatiles was noticeable in sensors number 3 and 8.

The capability of the PEN3 e-nose in distinguishing volatiles released from wood blocks of *Aesculus hippocastanum* decayed by different wood decay fungi were assessed using discrimination power values derived from pair-wise PCA as indicated in Table 8. Discrimination power indicates the proportion (expressed as a decimal fraction) of principle component identification elements within the analyte mixture that were detected and determined to be different in pairwise comparisons between analyte types (e.g., decay fungi-wood type combinations that produce unique decay types). Prerun tests using loadings analyses showed that sensor W5S and W2W contributed most to the discrimination by explaining more that 90% of the variance. Healthy undecayed control samples were geographically well discriminated. Healthy control wood of *Aesculus hippocastanum* were fairly well distinguished from wood decayed by *Armillaria mellea*, but less well discriminated from wood decayed by *Heterbasidion annosum*, and poorly discriminated from wood decayed by *Ganoderma lucidum* and *Inonotus dryadeus*. Good levels of discrimination also were found between *A. mellea* and *I. dryadeus* decay types, and between *I. dryadeus* and *H. annosum* decay types. All other pair-wise combinations of decay types were poorly discriminated.

**Figure 6.**
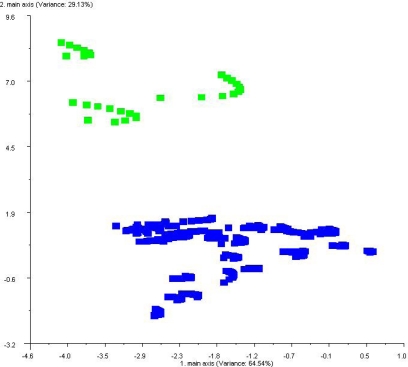
Discrimination of healthy (controls) and decayed (artificially inoculated) wood blocks samples by PCA based on differences in analyte principal components within headspace volatiles relative to standard deviations from the mean (Δσ). Color-coded labels are as follows: Green labels indicate volatiles from healthy, undecayed controls and blue labels indicate volatiles from decayed wood samples.

PCA mapping of volatiles from healthy and decayed wood based on statistical differences in standard deviations from the mean (Δσ). Figure 6 provides definitive evidence that uninoculated healthy control samples (green labels) were clearly separated from artificially-inoculated decayed wood blocks (blue labels). PCA analysis indicated that principal component 1 and principal component 2 explained 64.5% and 29.1%, respectively of the sample variance (σ^2^). Volatiles of healthy control wood samples were clustered in the upper left corner of the plot while decayed samples were clustered in the lower right portions of the 2-dimensional PCA plot.

**Figure 7.**
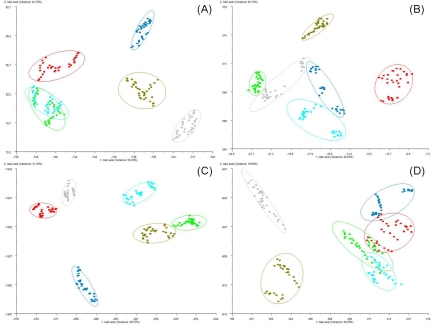

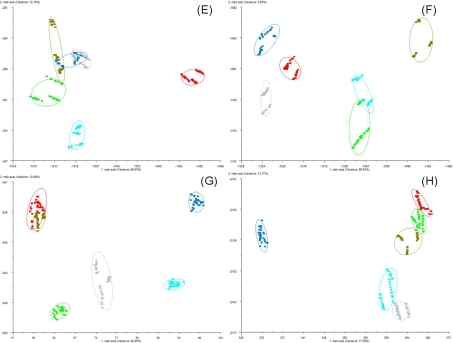
Aroma map plots based on LDA of volatiles from healthy and decayed wood blocks of (A) *Acer negundo*, (B) *Acer saccharinum*, (C) *Castanea sativa*, (D) *Cedrus deodara*, (E) *Celtis australis*, (F) *Platanus x acerifolia*, (G) *Quercus rubra* and (H) *Robinia pseudoacacia* after 12 months of decay by five different wood decay fungi defined by two principal components. Color codes for decay treatments, based on the wood decay fungus responsible for decay, are as follows: undecayed healthy wood (red); wood decayed by *Armillaria mellea* (green); wood decayed by *Ganoderma lucidum* (cyan); wood decayed by *Armillaria ostoyae* (dark blue); wood decayed by *Heterobasidion annosum* (olive); and wood decayed by *Inonotus dryadeus* (gray).

The possibility of recognizing the wood decay fungi species affecting the wood was investigated using the PEN3 e-nose as well. The discrimination between the different etiologic agents of decay was possible by examining the score plots provided by linear discriminant statistical analysis (LDA) of mean differences in standard deviations from the mean (Δσ). Volatile maps summarizing LDA of eight wood species after decay by five different wood decay fungi species, compared with healthy undecayed control woods, are presented in Figure 7 A–H.

Volatile signatures represented of healthy undecayed (control) plots were clustered in positions well separated from all volatile signatures of decayed woods for all wood species except for *Cedrus deodara* (Figure 7D) and *Quercus rubra* (Figure 7G). There was some slight overlap of cluster plots between healthy controls and wood decay by *Armillaria mellea* and *Armillaria ostoyae* for the *Cedrus deodara* wood type (Figure 7D). The healthy control plot for *Robinia pseudoacacia* wood species was very closely adjacent to the cluster plots of wood decayed by *Armillaria mellea*, but no overlap occurred between these plots (Figure 7D). Very little overlap of plots for decay types, caused by the five decay fungi, occurred for most wood species with the exception of some overlap between *Armillaria mellea* and *Ganoderma lucidum* in *Acer negundo* (Figure 7A), *Cedrus deodara* (Figure 7D) and *Platanus x acerifolia* (Figure 7F) wood types; and some overlap in the *Armillaria ostoyae* cluster plots with *Heterobasidion annosum and Inonotus dryadeus* in the *Celtis australis* wood type (Figure 7E).

## 4. Discussion and Conclusions

The AromaScan A32S e-nose was effective in the correct identification and discrimination of 93% of samples analyzed for different wood decay fungi in pure culture, but did not perform very well in terms of mean % aroma matches using pattern-recognition algorithms and reference libraries alone with small sample sizes. These poor match results (confidence levels) could be improved considerably by using greater replications of samples per sample type (aroma class) when creating recognition files using neural net training. Small sample sizes tend to result in very narrow representation of particular sample types that make sample pattern-recognition very difficult due to a limited database for comparisons with unknowns. Thus, recognition files should be constructed using local resident reference samples of sufficient numbers (at least five replications) for building recognition files to avoid geographical variations that are commonly associated with microbial strain differences, environmental conditions, and variations in host-wood composition resulting from metabolic differences in individual trees.

The AromaScan e-nose did, however, performed quite well using CPA to distinguish between healthy and incipient decayed wood of various decay fungus-host wood combinations tested. The good levels of % aroma class matches of 85% to 95% using reference library recognition files could be further improved with greater sample replications (sampling of unknowns from host woods) and lower levels of average error allowed in sample discrimination using the neural net recognition files. The current data analyses were made with recognition files set to an average error rate of approximately 0.20 to allow for greater variability in the data and reduce the rate of unknown determinations due to overtraining with the neural net when making recognition files. The AromaScan A32S performance results presented here were very similar to those reported by Wilson *et al*. [4], except that different decay fungus-host wood combinations were tested in some cases. Our results also were similar to those of Wilson *et al.* [21] for the discrimination of various healthy wood types derived from both hardwood and conifers. Thus, we confirm the capability and feasibility of this e-nose in providing effective discriminations for identifying and distinguishing healthy woody samples from those containing different agents of wood decay or decay types at incipient stages of decay.

The LibraNose 2.1 electronic nose performed exceptionally well in discriminating healthy wood from incipient decayed woods using PCA. Approximately 98–99% of the data variance could be explained by principal components 1 and 2 in all test cases. There were only four outlying data points for decayed wood samples that occurred outside of the majority cluster of data points for most of the decayed wood types, but no overlapping with the data-point cluster for healthy woods. These outlying decayed-wood data points are explained by differences in the VOC composition of headspace volatiles in certain host woods decayed by specific fungi. The most probably cause of this occurrence is the production of uniquely different secondary fungal metabolites (produced as a by-product of the wood decay process) by specific strains of *Armillaria ostoyae* on *Acer saccharinum* wood that are different from the normal compliment of fungal metabolites produced by the majority of the wood-rotting fungi tested. These outlying data much less likely are caused by unique volatile differences released from specific wood types because the wood in all cases consist of nonliving cells that have been baked in an oven prior to inoculation and thus do not have an active metabolism that could produce unique metabolites during the decay process. This e-nose had some difficulty discriminating between certain decay types in certain types of host woods. These problems potentially could be eliminated with the collection of new decay samples from host wood at a more advanced stage of decay. Most incidences of decay detection in real-life situations involve more advanced states of decay. The purpose of early detection of incipient decay is to provide time to assess the structural integrity of trees before they fail.

The PEN3 e-nose performed relatively poorly for discriminating healthy from decayed wood types for certain fungus-host combinations, but performed considerably better with other combinations depending on differences in headspace volatile composition between different sample types being compared. However, none of the discrimination comparisons of decayed woods versus healthy wood types indicated discrimination powers above 86% based on pair-wise PCA. By comparison, the discrimination power of the PEN3 instrument was much better (>98%) when distinguishing between eight different types of healthy woods [14]. The performance of this e-nose probably could be considerably improved using reference libraries and neural net training algorithms. Thus, this e-nose has good potential for distinguishing between decay types and healthy woods in field situations if application-specific reference libraries are established prior to analyses.

The purpose of this study was to independently test the capability of three e-noses, containing different detection mechanisms, to distinguish between healthy and decayed wood of various tree species. It was not possible to directly compare the performance of these three instruments using statistical methods because the methods available for data analysis were not exactly identical for each instrument due to differences in statistical algorithms used in the individual operating software that control each instrument. Thus, the performance of these three e-noses could only be compared based on their relative capabilities of discriminating healthy from decayed wood samples. The effective levels of sample discrimination gave some indication of the relative capabilities to detect decay in dead wood extracted from felled trees, but did not necessarily indicate that one e-nose was better than another for the purposes of detecting decay in standing trees that have active host-defence processes operating within the living sapwood. This determination will require more research involving the collection of field data directly from standing live trees. The LibraNose electronic nose provided the highest level of discrimination of the three e-nose types. However, the AromaScan conducting polymer e-nose, unlike the LibraNose with QMB sensors, had the greater advantage for identifying unknowns from diverse woody sample types due to the capability of utilizing prior-developed, application-specific reference libraries with pattern-recognition and neural-net training algorithms. The operating software supporting the PEN3 e-nose with MOS sensors also has the potential capability of utilizing reference libraries, but does not currently provide the option of using neural-net training algorithms. The capabilities established here of all three electronic noses in detecting differences between healthy and decayed wood derived from various tree species demonstrates the tentative feasibility of using electronic noses for the detection of incipient decay in standing trees. This evidence justifies further research to field-test these capabilities by the sampling of wood volatiles directly from living trees.

The very poor environmental conditions and stresses endured by trees planted in the urban environment have significantly decreased tree fitness, vigor and health due to increased exposure to attack by biotic pests, particularly wood decay fungi, and damage by various abiotic factors [14]. Conventional visual methods for assessing the external condition of urban trees are insufficient to indicate internal tree stability, and consequently require the repeated use of invasive and destructive field instruments by highly skilled personnel, yielding non-repeatable data that are very expensive to collect. The need for inexpensive alternative methods for assessing urban tree stability by relatively low-skilled personnel using portable electronic devices has prompted the current research to develop new methods using readily-available commercial electronic noses commonly utilized for many other applications in diverse industries [20]. The ultimate goal of this research is to generate data that could be used to produce recognition files within application-specific reference libraries of portable e-noses that would essentially automate the generation of information needed for the internal tree assessment process when wood volatiles taken from urban trees are analyzed. This could be achieved by producing reference libraries containing diagnostic volatile signature files of all possible decay types, based on decay volatile signatures of common combinations of the most damaging decay fungi-wood types found in urban trees that could be used for recognizing these specific types of decay in urban tree species. Specialized software could be developed to automatically search the reference library, following volatile acquisition by the e-nose of an extracted tree core sample, to determine whether specific types of decay and wood decay fungi are present in the living tree or if the tree is healthy. Previous research has demonstrated the capability of e-noses to discriminate between tree species based on volatiles analyzed from freshly collected or frozen tree wood-core samples [21], and to detect the presence of microbial plant pathogens, decay and disease in living trees [4]. The periodic collection of small, 5mm–diameter cylindrical tree cores using an increment borer is a relatively non-invasive method for collecting samples of internal woody tissues for decay detection, assessments and analysis. Most tree species are capable of forming callus tissues to close these small holes in trees over a relatively short period of time with little or no long-term effects.

Electronic noses are analytical chemical-sensing devices generally containing a sensor array capable of producing a digital aroma fingerprint of VOCs released from any source. Sensor sorption of VOCs is determined by the specific affinity of individual sensors to specific chemical types or classes. The capability of e-noses to discriminate between volatile aroma mixtures is dependent on not only the sensitivity of individual sensors to specific chemical species present in the analyte sample, but also on the relative quantities or molar ratios of chemicals present in the volatile mixture. In the current research, the chemical species released from healthy and decayed wood blocks consist of aroma mixtures derived from anabolic and catabolic biochemical processes of plant and/or fungal origin. The variability of chemical species present in these aroma mixtures, produced from a diverse assortment of metabolic pathways of the various wood decay fungi and host wood types (in response to the decay process), makes the electronic nose an ideal innovative tool for detecting the presence of decay or abnormal conditions resulting from decay or disease conditions in woody plant tissues of urban trees.

Commercial markets offer a wide variety of different electronic-nose devices that could potentially be used for the application of urban tree stability assessments. All three e-noses tested here provided effective discrimination of volatiles released from healthy and decayed wood types. However, our experiments suggest that polypyrrole-coated quartz microbalances with acoustic wave sensor arrays provide better results than other technologies (organic matrix-coated polymer-type and metal-oxide gas sensors) for detecting decay in wood and recognizing the presence of different wood decay fungi and types of decay in wood. The quartz microbalance e-nose provided significantly greater discrimination of volatile types released from woody samples when compared using PCA and LDA statistical analysis methods.

There are a number of potential logistic problems associated with the application of e-noses for decay and disease diagnoses in urban trees. The variability of ambient-air relative humidity and temperature in the urban environment can greatly affect readings of electronic noses due to the common sensitivities of sensor arrays to moisture and temperature variations. Other air contaminant factors that may affect the performance of electronic noses in the urban environment include the presence of air pollutants such as automobile exhaust and industrial effluents that could complicate data acquisition. These parameters may be controlled by preconditioning of reference air with various carbon and micro-particulate filters and moisture scrubbers followed by temperature regulation prior to injection into the sensor array. There is also a need to continue reducing the size and weight of mobile e-nose devices in order to make them more portable and easier to use. Improvements in miniaturization could be achieved by reducing the number of sensors present in the sensor array to the very minimum needed for effective discriminations in specific applications. Proper choices in sensor design to achieve sensor sensitivity specific to diagnostic chemical species (present only in decayed and healthy wood) should help to further optimize the number of sensors used in a portable sensor array. The variability of metabolites produced by different strains of the same decay fungi and individual trees of the same species can vary geographically under different environmental conditions. These variations require that the recognition files used in e-nose reference libraries be specific to the locality in which the instrument is used. The possibility of multiple decay types and fungi being present within the same tree may further complicate evaluations of decay types present in individual trees although determinations of decay vs. healthy wood should still be determinable in these cases. Software improvements will likely resolve some of the problems associated with difficult determinations. For all these reasons, further research is needed to evaluate the impact of urban factors that may affect the usefulness and reliability of electronic noses as decay- and disease-diagnostic tools for assessing urban tree health as well as other potential applications in urban and field situations.

The current research involved evaluations of the diagnostic capability of three distinct types of commercially-available electronic noses for discriminating between healthy and decayed wood samples. Similar samples could be extracted from urban trees to determine internal decay conditions and thus evaluate the stability of standing trees based on the presence and type of decays that may be present. The great quantity of data analyzed in the present study provides demonstrative evidence of the diagnostic feasibility of using electronic nose devices to assess the presence of incipient decay or wood decay fungi in wood colonized for relatively short time periods. Following additional research to solve logistic problems associated with these technologies, these instruments ultimately may serve as very effective and useful supporting or even stand-alone tools for the diagnosis of decays and early detection of attacks by wood decay fungi in urban trees.

## Figures and Tables

**Table 1.** Active matrix (metalloporphyrines) used to coat the quartz crystal microbalance sensors of LibraNose 2.1 e-nose [18].

**Sensor**	**Metalloporphorines**
1	Co-butoxytetraphenylporphyrine
2	Mn-butoxytetraphenylporphyrine
3	Cr-butoxytetraphenylporphyrine
4	Cu-butoxytetraphenylporphyrine
5	Ru-butoxytetraphenylporphyrine
6	Zn-butoxytetraphenylporphyrine
7	Sn-butoxytetraphenylporphyrine
8	Fe-butoxytetraphenylporphyrine

**Table 2.** Active matrix (polypirrole polymers) used to coat the quartz crystal microbalance sensors of LibraNose 2.2 e-nose [18].

**Sensor**	**Aldehydes**	**Polymers**
1	Phenanthrene-9-aldehyde	Poly[2-(9-phenanthrylmethyl)]-1*H*-pyrrole
2	*trans*-Cinnamaldehyde	Poly{2-[2-(2E)-3-phenylprop-2-enyl]}-1*H*-pyrrole
3	Ferrocene carboxaldehyde	Poly(ferrocene)-1*H*-pyrrole
4	Benzaldehyde	Poly[2-(benzyl)]-1*H*-pyrrole
5	Anisaldehyde	Poly[2-4(methoxybenzyl)]-1*H*-pyrrole
6	3-Hydroxy-4-methoxybenzaldehyde	Poly[2-ethoxy-5-(1H-pyrrol-2-ylmethyl)]phenol
7	Thiophene-2-carboxyaldehyde	Poly[2-(thien-2-ylmethyl)]-1*H*-pyrrole
8	nd	nd

**Table 3.** Sensor sensitivity of individual sensors within the sensor array of the PEN3 e-nose.

**Sensor number**	**Sensor name**	**Sensor sensitivity and general description**	**Sensitivity levels**
1	W1C	Aromatic compounds	Toluene, 10 mg kg^−1^
2	W5S	Very sensitive, broad range of sensitivity, reacts to nitrogen oxides, very sensitive with negative signals	NO_2_, 1 mg kg^−1^
3	W3C	Ammonia, used as sensor for aromatic compounds	Benzene, 10 mg kg^−1^
4	W6S	Mainly hydrogen	H_2_, 0,1 mg kg^−1^
5	W5C	Alkanes, aromatic compounds, less polar compounds	Propane, 1 mg kg^−1^
6	W1S	Sensitive to methane. Broad range.	CH_3_, 100 mg kg^−1^
7	W1W	Reacts to sulphur compounds, H_2_S. Otherwise sensitive to many terpenes and sulphur-containing organic compounds.	H_2_S, 1 mg kg^−1^
8	W2S	Detects alcohol partially aromatic compounds, broad range	CO, 100 mg kg^−1^
9	W2W	Aromatic compounds, sulphur organic compounds	H_2_S, 1 mg kg^−1^
10	W3S	Reacts to high concentrations (>100 mg/kg) of methane-aliphatic compounds	Not determined

**Table 4.** Tests of the capability of the AromaScan A32S e-nose to identify 24 wood decay fungi in pure agar culture using conductive polymer analysis with recognition files constructed from a fungus-specific reference library.

**Decay fungi unknowns**	**N=**	**Correctly identified^1^**	**Incorrectly identified^2^**	**Indeterminate, not identified^3^**	**Mean % aroma class match^4^**
*Amylostereum areolatum*	2	2	0	0	73.8
*Armillaria gallica*	1	1	0	0	84.5
*Armillaria mellea*	2	2	0	0	91.9
*Armillaria ostoyae*	1	1	0	0	66.9
*Armillaria tabescens*	1	1	0	0	75.8
*Cerrena unicolor*	2	2	0	0	73.4
*Daedalea quercina*	1	1	0	0	71.5
*Daldinia concentrica*	4	4	0	0	85.3
*Fomitopsis officinalis*	1	1	0	0	67.1
*Ganoderma lucidum*	4	4	0	0	91.0
*Gloeophyllum sepiarium*	1	1	0	0	82.4
*Hericium erinaceus*	3	3	0	0	73.1
*Heterobasidion annosum*	1	1	0	0	77.9
*Inonotus dryadeus*	1	0	0	1	Unk
*Laetiporus sulphureus*	1	1	0	0	75.0
*Oxyporus latemarginatus*	5	5	0	0	76.2
*Phanerochaete gigantea*	1	1	0	0	100.0
*Phellinus pini*	2	0	0	2	Unk
*Pleurotus ostreatus*	1	1	0	0	64.5
*Postia caesia*	1	1	0	0	73.4
*Spongipellis pachyodon*	2	2	0	0	86.1
*Stereum hirsutum*	2	2	0	0	63.0
*Wolfiporia cocos*	1	1	0	0	66.0
*Xylaria polymorpha*	3	3	0	0	69.5
**Totals**	**44**	**41 (93.2%)**	**0**	**3 (6.8%)**	

1 Number of unknown wood decay fungi samples correctly identified in pure 4.5% MA cultures.

2 Number of unknown wood decay fungi samples identified to an incorrect aroma class.

3 Number of unknown wood decay fungi samples not identified to an aroma class due to <70% ownership in any one aroma class.

4 Mean percentage match of unknown wood decay fungi samples to the correct aroma class identity. Unk = unknown, *i.e.*, sampled was not identified.

**Table 5.** Tests of the AromaScan A32S in the identifications of eight uninoculated healthy wood types by conductive polymer analysis using recognition files constructed from a wood-specific reference library.

**Wood type unknowns**	**n=**	**Correctly identified^1^**	**Incorrectly identified^2^**	**Indeterminate, not identified^3^**	**Mean % aroma class match^4^**
*Fraxinus pennsylvanica*	5	4	0	1	91.7
*Liquidambar styraciflua*	5	3	1	1	78.4
*Pinus taeda*	5	4	0	1	75.3
*Populus deltoides*	5	4	1	0	91.4
*Quercus lyrata*	5	5	0	0	94.5
*Quercus nuttallii*	5	5	0	0	95.2
*Taxodium distichum*	5	3	2	0	85.5
*Thuja occidentalis*	5	4	0	1	92.7
**Totals**	**40**	**32 (80.0%)**	**4 (10.0%)**	**4 (10.0%)**	

1 Number of unknown healthy wood type samples correctly identified in pure 4.5% MA cultures.

2 Number of unknown healthy wood type samples identified to an incorrect aroma class.

3 Number of unknown healthy wood type samples not identified to an aroma class due to <70% ownership in any one aroma class.

4 Mean percentage match of unknown healthy wood type samples to the correct aroma class identity.

**Table 6.** Tests of the AromaScan A32S for the identification of 11 wood decay types, associated with specific wood-rotting fungi, determined by conductive polymer analysis using recognition files constructed from decay and host-specific reference libraries. Nine different wood types (species) were tested for each decay type, defined by the fungus causing the decay, as follows: *Fraxinus pennsylvanica*; *Liquidambar styraciflua*; *Populus deltoide*s; *Plantanus occidentalis*; *Pinus taeda*; *Quercus nuttallii*; *Quercus lyrata*; *Taxodium distichum*; and *Thuja occidentalis*.

**Decay type unknowns**	**n=**	**Correctly identified^1^**	**Incorrectly identified**	**Indeterminate, not identified^2^**	**Mean % aroma class match^3^**
*Armillaria gallica*	27	26	1	0	88.4
*Armillaria mellea*	54	51	0	3	95.1
*Armillaria ostoyae*	27	26	0	1	89.1
*Armillaria tabescens*	27	26	0	1	89.3
*Daedalea quercina*	27	26	0	1	88.4
*Ganoderma lucidum*	54	47	1	6	86.4
*Heterobasion annosum*	27	27	0	0	88.0
*Inonotus dryadeus*	27	26	0	1	88.4
*Laetiporus sulphureus*	27	24	3	0	85.0
*Phellinus pini*	54	53	0	1	95.6
*Stereum hirsutum*	27	27	0	0	88.9
**Totals**	**378**	**359 (95.0%)**	**5 (1.3%)**	**14 (3.7%)**	

1 Number of unknown decay type samples correctly identified in pure 4.5% MA cultures.

2 Number of unknown decay type samples identified to an incorrect aroma class.

3 Number of unknown decay type samples not identified to an aroma class due to <70% ownership in any one aroma class.

4 Mean percentage match of unknown decay type samples to the correct aroma class identity.

**Table 7.** Three-dimensional PCA pair-wise comparison of headspace volatile metabolites released from *Tilia* sp. wood blocks decayed by different wood decay fungi (*Armillaria mellea*, *A. ostoyae*, *Ganoderma lucidum*, *Heterobasidion annosum*, *Inonotus dryadeus*) relative to undecayed healthy (control) wood blocks.

**Analyte 1 volatiles**	**Analyte 2 volatiles**	**Quality factor significance^1^**
Undecayed healthy *Tilia* sp.	*Tilia* sp. decayed by *A. mellea*	13,527 ***
	*Tilia* sp. decayed by *A. ostoyae*	9,832 ***
	*Tilia* sp. decayed by *G. lucidum*	27,957 ****
	*Tilia* sp. decayed by *H. annosum*	51,873 ****
	*Tilia* sp. decayed by *I. dryadeus*	3,567 **

1 Statistical levels of difference between analyte aroma classes, indicated by quality factor significance according to 3-d PCA, are based on the following approximate scale of significant differences: * = P < 0.01, ** = P < 0.001, *** = P < 0.0001; **** = P < 0.00001, respectively. Quality factor (QF) is a numerical value that provides an absolute measurement of the discrimination (statistical difference) between two sample types, being compared using PCA, by indicating the distance between the centers of the data clusters being compared relative to the spread distribution of those two clusters. A quality factor of 2 or more is considered a significant discrimination at an approximately statistical significance level of P = 0.10.

**Table 8.** The discrimination power of the PEN3 e-nose for distinguishing decay of *Aesculus hippocastanum* wood blocks, caused by four different species of wood decay fungi, based on PCA by pair-wise comparisons of headspace volatiles. Principal component 1 and principal component 2 explained 65.2% and 28.9%, respectively of the sample variance (σ^2^) in the data. The data values indicate the decimal fraction overlap of principal component aroma elements that the sample analytes share in common. By definition, a specific analyte shares all principal components when compared against itself and the theoretical decimal fraction overlap is 1.000 for self-comparisons.

**Analyte type**	**Undecayed healthy control**	***Armillaria mellea***	***Inonotus dryadeus***	***Ganoderma lucidum***	***Heterobasidion annosum***
**Undecayed healthy control**		0.801	0.023	0.529	0.793
***Armillaria mellea***	0.801		0.824	0.509	0.767
***Inonotus dryadeus***	0.023	0.824		0.620	0.857
***Ganoderma lucidum***	0.529	0.509	0.620		0.748
***Heterobasidion annosum***	0.793	0.767	0.857	0.749	

## Footnotes

1 Color codes for decay treatments, based on the wood decay fungus responsible for decay, are as follows: *Armillaria mellea* (red); *Armillaria ostoyae* (gray); *Ganoderma lucidum* (blue); *Heterobasidion annosum* (magenta); *Inonotus dryadeus* (green); and healthy, undecayed control (yellow).
